# Derivation and Validation of a New Disease Activity Assessment Tool With Higher Accuracy for Takayasu Arteritis

**DOI:** 10.3389/fimmu.2022.925341

**Published:** 2022-06-17

**Authors:** Oh Chan Kwon, Min-Chan Park

**Affiliations:** Division of Rheumatology, Department of Internal Medicine, Yonsei University College of Medicine, Seoul, South Korea

**Keywords:** Takayasu arteritis, vasculitis, disease activity, assessment, accuracy

## Abstract

**Objective:**

To develop a new disease activity assessment tool with high accuracy for Takayasu arteritis.

**Methods:**

Individual items from National Institute of Health (NIH) criteria and the Indian Takayasu Clinical Activity Score (ITAS2010) were tested as candidate variables to develop a new disease activity assessment tool in a derivation cohort (N = 100). Physician global assessment on disease activity was used as the gold standard. Multivariable logistic regression models were constructed and the model with the highest accuracy was identified. A formula assessing disease activity was generated using simplified β coefficients (rounded to decimal place). Diagnostic performance was evaluated through estimating the area under the curve (AUC). The new assessment tool was subsequently validated in a validation cohort (N = 46).

**Results:**

The multivariable model yielding the highest accuracy consisted of a high erythrocyte sedimentation rate (ESR), NIH criteria 1 and 4, and carotidynia. Using simplified β coefficients, the following disease activity assessment tool was developed: high ESR (3 points), NIH criterion 1 (2 points), NIH criterion 4 (4 points), and carotidynia (3 points) (total score ≥5, active; total score <5, inactive). The new disease activity assessment tool had a higher AUC (89.37) for discriminating active and inactive diseases than NIH criteria (AUC 77.96), ITAS2010 (AUC 66.12), ITAS-ESR (AUC 75.58), and ITAS-C-reactive protein (AUC 71.34). The AUC (85.23) of the new assessment tool was similar in the validation cohort.

**Conclusion:**

A new disease activity assessment tool that consists of high ESR, NIH criteria 1 and 4, and carotidynia had higher accuracy in discriminating active and inactive disease than currently used clinical assessment tools.

## Introduction

Takayasu arteritis (TAK) is a chronic inflammatory disease that causes granulomatous inflammation of the aorta and its major branches (1). Vessel inflammation results in irreversible structural damage, such as stenosis or aneurysm formation (2). For the treatment of TAK, interrupting active vessel inflammation before structural vessel damage occurs is crucial (3, 4). For the timely interruption of active vessel inflammation, accurate assessment of disease activity is important. However, assessing disease activity in TAK is challenging as neither clinical symptoms nor laboratory data accurately reflect actual inflammation of the arterial wall (3, 5, 6).

Several clinical assessment tools for assessing disease activity in TAK have been developed. National Institute of Health (NIH) criteria, which consist of systemic symptoms, the erythrocyte sedimentation rate (ESR), vascular symptoms, and angiographic features, were the first tools used to assess disease activity in TAK (3). However, NIH criteria are suboptimal in detecting pathologically proven active disease (7). The Birmingham Vasculitis Activity Score (BVAS), which is a validated tool for assessing disease activity in small and medium vessel vasculitis (8), has also been used to assess disease activity in TAK (9). However, as the BVAS is a tool originally developed for small and medium vessel vasculitis, the value of the BVAS for assessing disease activity of TAK is limited as most of the 11 organ systems included in the BVAS are not affected in TAK (10). The disease extent index for TAK (DEI.Tak) is another assessment tool used for patients with TAK, which was created using the BVAS as a template (11). The DEI.Tak included rarely used items while not taking into account acute phase reactants or imaging findings (11), and it has not been widely accepted (12). The most recently developed disease activity assessment tool is the Indian Takayasu Clinical Activity Score (ITAS2010), which is derived from the DEI.Tak (13). It scores clinical features newly developed in the previous three months, with an additional version that includes acute-phase reactants (ITAS-A) (13). Although the ITAS scoring system has been found to be discriminatory for activity, imaging findings are not included in this scoring system, and studies have shown unsatisfactory agreement between the ITAS and the physician global assessment (PGA) (12, 14).

Given the lack of an accurate disease activity assessment tool that can be widely adopted for use in research or clinical practice, we aimed to develop a new disease activity assessment tool that is highly accurate, using the PGA as the gold standard.

## Patients and Methods

### Patients

Patients with TAK who underwent laboratory tests and imaging studies [computed tomography (CT) scans] for the purpose of disease activity assessment between 2012 and 2021 at two referral hospitals were retrospectively included for analysis. All patients fulfilled the 1990 American College of Rheumatology criteria for the classification of TAK (15). Patients were randomly assigned to either a derivation or a validation cohort. Data concerning the following at the time of disease activity assessment were reviewed: age, sex, disease duration, type of vascular involvement according to the Hata classification (16), involvement of the pulmonary artery and renal artery, ESR (measured by Test 1 [Alifax, Padova, Italy]; cut-off value for high ESR was >20 mm/h for female, and >15 mm/h for male), C-reactive protein (CRP) level (cut-off value for high CRP was >6 mg/L), total scores of disease activity assessment tools including NIH criteria, ITAS2010, ITAS-ESR, and ITAS-CRP, fulfilment of individual items from NIH criteria (NIH criterion 1, new onset or worsening of systemic features, such as fever or musculoskeletal symptoms; NIH criterion 2, new onset or worsening of elevated ESR; NIH criterion 3, new onset or worsening of features of vascular ischemia or inflammation, such as claudication, diminished or absent pulse, bruit, carotidynia, asymmetric blood pressure in either upper or lower limbs; and NIH criterion 4, new onset or worsening of typical angiographic features) (3) and the ITAS2010, PGA (active disease or inactive disease), and the use of a glucocorticoid (none-to-low dose, ≤7.5 mg of prednisolone or equivalent/day; or medium-to-high dose, >7.5 mg of prednisolone or equivalent/day) (17), methotrexate (yes or no), and azathioprine (yes or no). Fulfilment of NIH criterion 4 was assessed based on a CT scan. The NIH criterion 4 was considered fulfilled if one or more of the following findings were observed in the CT scans: (i) new luminal vascular lesions in previously unaffected arterial territory; (ii) progression of a previous luminal vascular lesion; and (iii) presence of concentric arterial wall thickening with delayed enhance.

This study was approved by the Institutional Review Board (IRB) of Gangnam Severance Hospital (IRB No: 3-2021-0445). Owing to this study’s retrospective design, the requirement for informed consent was waived.

### The PGA

The PGA on disease activity (active or inactive disease) was used as the gold standard. The PGA was determined by the treating physician after laboratory and imaging tests had been performed. Therefore, the PGA was comprehensively based on patient’s symptoms, acute phase reactants, and imaging findings. Active disease was defined as presence of two or more of the following: (i) carotidynia; (ii) ischemic episodes; (iii) new bruit or asymmetry in pulse or blood pressure; (iv) constitutional systemic symptoms such as fever, malaise, weight loss, or musculoskeletal symptoms; and (v) elevated ESR and/or CRP. If new or progression of vascular lesions (luminal or arterial wall thickening) were detected on CT scan, presence of one or more of the above was considered as active disease. Constitutional systemic symptoms or elevated acute phase reactants in the absence of any clinical feature directly attributable to vasculitis were not considered as active disease.

### Statistical Analysis

The sample size was calculated based on the area under the curve (AUC) of the new disease activity assessment tool. A difference of 0.15 between an AUC of 0.7, which is generally considered as an acceptable accuracy, and the new disease activity assessment tool with an AUC of 0.85 was selected as the minimum clinically significant value. We estimated that a sample size of 82 patients would be sufficient to evaluate the outcome at a significance level of 0.05 (two-sided) with 80% power. Patients were assigned to the derivation cohort and validation cohort in a 7:3 ratio.

Continuous variables following normal or non-normal distribution are expressed as mean [± standard deviation (SD)] or median [interquartile range (IQR)], respectively, and categorical variables are expressed as numbers (%).

To develop a new disease activity assessment tool, we first conducted univariable logistic regression analyses in the derivation cohort using the PGA (active or inactive disease) as the dependent variable. We considered all individual items in NIH criteria and the ITAS2010 as potential components of the new disease activity assessment tool. Therefore, each item was used as an independent variable in the univariable logistic regression analysis. Variables that were statistically significant in the univariable logistic regression analyses were selected for multivariable stepwise logistic regression analysis. Taking multicollinearity among the variables into account, eight different multivariable models were constructed. Among the eight multivariable models, the model that yielded the highest AUC for distinguishing active disease from inactive disease was selected and used to develop the new disease activity assessment tool. The new disease activity assessment tool formula was obtained by multiplying each variable by its simplified β coefficient (β coefficient rounded to decimal place) and then summing the results.

Receiver operating characteristic (ROC) curve analysis was performed to determine the cut-off value of the new disease activity assessment tool that best discriminated active disease and inactive disease. The cut-off value was determined at the value where the Youden index was maximum. Diagnostic performance of the new disease activity assessment tool was evaluated by estimating AUC, sensitivity, specificity, accuracy, positive predictive value (PPV), and negative predictive value (NPV) with their respective 95% confidence intervals (95% CIs). The diagnostic performance of the new disease activity assessment tool was compared with that of NIH criteria, the ITAS2010, the ITAS-ESR and the ITAS-CRP.

For validation of the new disease activity assessment tool developed in the derivation cohort, AUC, sensitivity, specificity, accuracy, PPV, and NPV with their respective 95% CIs were estimated in the validation cohort.

A *P-*value of <0.05 was considered statistically significant. All analyses were conducted using SAS (version 9.4, SAS Inc., Cary, NC, USA) software.

## Results

### Patients’ Characteristics

A total of 146 patients with TAK were included (100 patients in the derivation cohort and 46 patients in the validation cohort). In the derivation cohort, the mean age of the patients was 43.3 ( ± 14.5) years, and 89.0% were female. The median ESR, CRP, NIH criteria, ITAS2010, ITAS-ESR, and ITAS-CRP values were 31.0 (16.3–55.0) mm/h, 2.2 (1.0–11.3) mg/L, 1.0 (1.0–2.0), 1.5 (0.0–3.0), 3.0 (1.0–4.0), and 2.0 (1.0–4.0), respectively. Proportions of patients with high ESR and high CRP levels were 67.0% and 35.0%, respectively. According to the PGA, 46 (46.0%) and 54 (54.0%) patients had active disease and inactive disease, respectively. Details of patient characteristics in the derivation cohort are shown in **Table 1**.

**Table 1:** Characteristics of the patients at the time of disease activity assessment.

	Derivation cohort (N = 100)	Validation cohort (N = 46)
Age, years, mean ( ± SD)	43.3 ± 14.5	41.7 ± 15.5
Female sex, n (%)	89 (89.0)	38 (82.6)
Disease duration, years, median (IQR)	1.2 (1.0–5.9)	1.5 (1.0–5.9)
Type of vascular involvement, n (%)		
I	17 (17.0)	13 (28.3)
IIA	15 (15.0)	3 (6.5)
IIB	16 (16.0)	9 (19.6)
III	0 (0.0)	2 (4.3)
IV	4 (4.0)	2 (4.3)
V	48 (48.0)	17 (37.0)
Pulmonary artery involvement, n (%)	9 (9.0)	5 (10.9)
Renal artery involvement, n (%)	13 (13.0)	13 (28.3)
Use of glucocorticoid, n (%)		
None-to-low dose	94 (94.0)	39 (84.8)
Medium-to-high dose	6 (6.0)	7 (15.2)
Use of methotrexate, n (%)	16 (16.0)	10 (21.7)
Use of azathioprine, n (%)	7 (7.0)	2 (4.3)
ESR, mm/h, median (IQR)	31.0 (16.3–55.0)	31.5 (17.0–83.3)
High ESR, n (%)	67 (67.0)	31 (67.4)
CRP, mg/L, median (IQR)	2.2 (1.0–11.3)	3.4 (1.0–24.6)
High CRP, n (%)	35 (35.0)	18 (39.1)
NIH criteria, median (IQR)	1.0 (1.0–2.0)	1.0 (1.0–2.0)
NIH criterion 1, n (%)	20 (20.0)	8 (17.4)
NIH criterion 2, n (%)	56 (56.0)	27 (58.7)
NIH criterion 3, n (%)	27 (27.0)	10 (21.7)
NIH criterion 4, n (%)	25 (25.0)	13 (28.3)
ITAS2010, median (IQR)	1.5 (0.0–3.0)	1.0 (0.0–3.0)
ITAS-ESR, median (IQR)	3.0 (1.0–4.0)	2.5 (2.0–4.3)
ITAS-CRP, median (IQR)	2.0 (1.0–4.0)	2.0 (1.0–4.0)
Malaise or weight loss, n (%)	12 (12.0)	1 (2.2)
Myalgia or arthralgia or arthritis, n (%)	16 (16.0)	5 (10.9)
Headache, n (%)	10 (10.0)	5 (10.9)
Severe abdominal pain, n (%)	9 (9.0)	3 (6.5)
Abortions, n (%)	0 (0.0)	0 (0.0)
Diastolic BP >90 mmHg, n (%)	10 (10.0)	5 (10.9)
Systolic BP >120 mmHg, n (%)	21 (21.0)	15 (32.6)
Stroke, n (%)	4 (4.0)	2 (4.4)
Seizure, n (%)	0 (0.0)	0 (0.0)
Syncope, n (%)	0 (0.0)	0 (0.0)
Vertigo or dizziness, n (%)	6 (6.0)	5 (10.9)
Bruits, n (%)	2 (2.0)	2 (4.4)
Pulse inequality, n (%)	10 (10.0)	4 (8.7)
Loss of pulse, n (%)	7 (7.0)	1 (2.2)
Claudication, n (%)	7 (7.0)	4 (8.7)
Carotidynia, n (%)	12 (12.0)	5 (10.9)
Aortic incompetence, n (%)	11 (11.0)	7 (15.2)
Myocardial infarct or angina, n (%)	3 (3.0)	0 (0.0)
Cardiomyopathy or cardiac failure, n (%)	3 (3.0)	2 (4.4)
PGA, n (%)		
Active	46 (46.0)	24 (52.2)
Inactive	54 (54.0)	22 (47.8)

BP, blood pressure; CRP, C-reactive protein; ESR, erythrocyte sedimentation rate; ITAS2010, Indian Takayasu Clinical Activity Score; NIH, National Institute of Health; PGA, physician global assessment.

### Development of a New Disease Activity Assessment Tool

In the derivation cohort, results of the univariable logistic regression analysis showed ESR (continuous variable; *P* < 0.001), high ESR (categorical variable, yes or no; *P* < 0.001), CRP (continuous variable; *P* = 0.020), high CRP (categorical variable, yes or no; *P* = 0.005), NIH criterion 1 (*P* = 0.020), NIH criterion 3 (*P* = 0.014), NIH criterion 4 (*P* < 0.001), malaise or weight loss (*P* = 0.043), carotidynia (*P* = 0.014), and aortic incompetence *(P* = 0.023) as variables significantly associated with active disease (**Table 2**).

**Table 2:** Comparison of individual items constituting disease activity assessment tools between active and inactive patients in the derivation cohort according to the PGA.

	Active disease (N = 46)	Inactive disease (N = 54)	Unadjusted OR (95% CI)	*P-*value
ESR, mm/h, median (IQR)	43.0 (27.5–75.3)	20.0 (10.0–37.3)	1.03 (1.02–1.05)	< 0.001
High ESR, n (%)	41 (89.1)	26 (48.2)	8.83 (3.03–25.76)	< 0.001
CRP, mg/L, median (IQR)	6.1 (1.8–22.1)	1.2 (0.8–3.9)	1.04 (1.01–1.08)	0.020
High CRP, n (%)	23 (50.0)	12 (22.2)	3.50 (1.48–8.30)	0.005
NIH criterion 1, n (%)	14 (30.4)	6 (11.1)	3.50 (1.22–10.06)	0.020
NIH criterion 2, n (%)	29 (63.0)	27 (50.0)	1.71 (0.77–3.80)	0.192
NIH criterion 3, n (%)	18 (39.1)	9 (16.7)	3.21 (1.27–8.14)	0.014
NIH criterion 4, n (%)	23 (50.0)	2 (3.7)	21.00 (5.09–86.61)	< 0.001
Malaise or weight loss, n (%)	9 (19.6)	3 (5.6)	4.14 (1.05–16.33)	0.043
Myalgia or arthralgia or arthritis, n (%)	10 (21.7)	6 (11.1)	2.22 (0.74–6.68)	0.155
Headache, n (%)	7 (15.2)	3 (5.6)	3.05 (0.74–12.56)	0.123
Severe abdominal pain, n (%)	6 (13.0)	3 (5.6)	2.55 (0.60–10.83)	0.205
Abortions, n (%)	0 (0.0)	0 (0.0)	N/A	N/A
Diastolic BP >90 mmHg, n (%)	5 (10.9)	5 (9.3)	1.20 (0.32–4.42)	0.789
Systolic BP >120 mmHg, n (%)	6 (13.0)	15 (27.8)	0.39 (0.14–1.11)	0.077
Stroke, n (%)	2 (4.4)	2 (3.7)	1.18 (0.16–8.74)	0.870
Seizure, n (%)	0 (0.0)	0 (0.0)	N/A	N/A
Syncope, n (%)	0 (0.0)	0 (0.0)	N/A	N/A
Vertigo or dizziness, n (%)	2 (4.4)	4 (7.4)	0.57 (0.10–3.25)	0.526
Bruits, n (%)	0 (0.0)	2 (3.7)	0.23 (0.01–9.50)	0.436
Pulse inequality, n (%)	6 (13.0)	4 (7.4)	1.88 (0.50–7.10)	0.355
Loss of pulse, n (%)	5 (10.9)	2 (3.7)	3.17 (0.59–17.17)	0.181
Claudication, n (%)	6 (13.0)	1 (1.9)	7.95 (0.92–68.63)	0.060
Carotidynia, n (%)	10 (21.7)	2 (3.7)	7.22 (1.49–34.94)	0.014
Aortic incompetence, n (%)	9 (19.6)	2 (3.7)	6.32 (1.29–30.98)	0.023
Myocardial infarct or angina, n (%)	3 (6.5)	0 (0.0)	8.77 (0.28–274.53)	0.217
Cardiomyopathy or cardiac failure, n (%)	1 (2.2)	2 (3.7)	0.58 (0.05–6.59)	0.659

BP, blood pressure; CI, confidence interval; CRP, C-reactive protein; ESR, erythrocyte sedimentation rate; N/A, not applicable; NIH, National Institute of Health; OR, odds ratio; PGA, physician global assessment.

Using these variables, eight multivariable models were constructed (**Table 3**). Among the eight models, model 6 yielded the highest AUC (89.49). Model 6 included high ESR, high CRP, NIH criterion 1, NIH criterion 4, carotidynia, and aortic incompetence as independent variables; high ESR, NIH criterion 1, NIH criterion 4, and carotidynia remained as statistically significant in the final model. This model was selected for the development of the new disease activity assessment tool. The β coefficients of high ESR, NIH criterion 1, NIH criterion 4, and carotidynia were 2.77 (simplified β: 3), 1.53 (simplified β: 2), 3.63 (simplified β: 4), and 3.37 (simplified β: 3), respectively (**Table 4**). Using simplified β coefficients of each variable, we generated a new disease activity assessment tool as follows: high ESR (3 points), NIH criterion 1 (2 points), NIH criterion 4 (4 points), and carotidynia (3 points) (total score ≥5, active; total score <5, inactive). The glossary of the terms included in the assessment tool is as follows: high ESR, >20 mm/h for female, and >15 mm/h for male; NIH criterion 1, new onset or worsening of systemic features, such as fever or musculoskeletal symptoms; NIH criterion 4, one or more of the following findings observed in the CT scans, (i) new luminal vascular lesions in previously unaffected arterial territory, (ii) progression of a previous luminal vascular lesion, and (iii) presence of concentric arterial wall thickening with delayed enhance; and carotidynia, tenderness or pain during palpation of the carotid arteries.

**Table 3:** Multivariable models using stepwise regression.

	Model 1		Model 2		Model 3		Model 4	
	Adjusted OR (95% CI)	*P-*value	Adjusted OR (95% CI)	*P-*value	Adjusted OR (95% CI)	*P*-value	Adjusted OR (95% CI)	*P-*value
ESR	1.04 (1.02–1.06)	<0.001	1.04 (1.02–1.06)	<0.001	1.04 (1.02–1.06)	<0.001	1.04 (1.02–1.06)	<0.001
High ESR								
CRP								
High CRP								
NIH criterion 1								
NIH criterion 3	3.71 (1.12–12.26)	0.032			3.71 (1.12–12.26)	0.032		
NIH criterion 4	21.87 (4.41–108.39)	<0.001	28.52 (5.48–148.35)	<0.001	21.87 (4.41–108.39)	<0.001	28.52 (5.48–148.35)	<0.001
Malaise or weight loss								
Carotidynia			14.81 (2.35–93.46)	0.004			14.81 (2.35–93.46)	0.004
Aortic incompetence								
	Model 5		Model 6		Model 7		Model 8	
	Adjusted OR (95% CI)	*P-*value	Adjusted OR (95% CI)	*P-*value	Adjusted OR (95% CI)	*P-*value	Adjusted OR (95% CI)	*P-*value
ESR								
High ESR	10.20 (2.65–39.23)	<0.001	15.92 (2.87–88.49)	0.002	10.20 (2.65–39.23)	<0.001	17.26 (3.32–89.68)	<0.001
CRP								
High CRP								
NIH criterion 1			4.60 (1.23–17.26)	0.024				
NIH criterion 3								
NIH criterion 4	29.60 (5.40–162.16)	<0.001	37.63 (5.83–243.10)	<0.001	29.60 (5.40–162.16)	<0.001	34.85 (5.37–226.34)	<0.001
Malaise or weight loss								
Carotidynia			29.18 (2.87–296.30)	0.004			19.97 (2.07–192.71)	0.010
Aortic incompetence								

AUC, Area under the curve; CI, confidence interval; CRP, C-reactive protein; ESR, erythrocyte sedimentation rate; NIH, National Institute of Health; OR, odds ratio.

Model 1 included ESR, CRP, NIH criterion 1, NIH criterion 3, NIH criterion 4, and aortic incompetence (AUC, 88.124).

Model 2 included ESR, CRP, NIH criterion 1, NIH criterion 4, carotidynia, and aortic incompetence (AUC, 88.4058).

Model 3 included ESR, CRP, NIH criterion 3, NIH criterion 4, malaise or weight loss, and aortic incompetence (AUC, 88.124).

Model 4 included ESR, CRP, NIH criterion 4, malaise or weight loss, carotidynia, and aortic incompetence (AUC, 88.4058).

Model 5 included High ESR, high CRP, NIH criterion 1, NIH criterion 3, NIH criterion 4, and aortic incompetence (AUC, 82.8905).

Model 6 included High ESR, high CRP, NIH criterion 1, NIH criterion 4, carotidynia, and aortic incompetence (AUC, 89.4928).

Model 7 included High ESR, high CRP, NIH criterion 3, NIH criterion 4, malaise or weight loss, and aortic incompetence (AUC, 82.8905).

Model 8 included High ESR, high CRP, NIH criterion 4, malaise or weight loss, carotidynia, and aortic incompetence (AUC, 85.5072).

**Table 4:** Development of new disease activity assessment tool.

	β (SE)	Adjusted OR (95% CI)	*P-*value	Simplified β
High ESR	2.77 (0.88)	15.92 (2.87–88.49)	0.002	3
NIH criterion 1	1.53 (0.67)	4.60 (1.23–17.26)	0.024	2
NIH criterion 4	3.63 (0.95)	37.63 (5.83–243.10)	<0.001	4
Carotidynia	3.37 (1.18)	29.18 (2.87–296.30)	0.004	3

CI, confidence interval; ESR, erythrocyte sedimentation rate; NIH, National Institute of Health; OR, odds ratio; SE, standard error.

New disease activity assessment tool: high ESR (3 points), NIH criterion 1 (2 points), NIH criterion 4 (4 points), and carotidynia (3 points) (total score ≥5, active; total score <5, inactive).

High ESR: >20 mm/h for female, and >15 mm/h for male.

NIH criterion 1: New onset or worsening of systemic features, such as fever or musculoskeletal symptoms.

NIH criterion 4: One or more of the following findings observed in the CT scans, (i) new luminal vascular lesions in previously unaffected arterial territory; (ii) progression of a previous luminal vascular lesion; and (iii) presence of concentric arterial wall thickening with delayed enhance.

Carotidynia: Tenderness or pain during palpation of the carotid arteries.

### Diagnostic Performance of the New Disease Activity Assessment Tool

**Figure 1:**
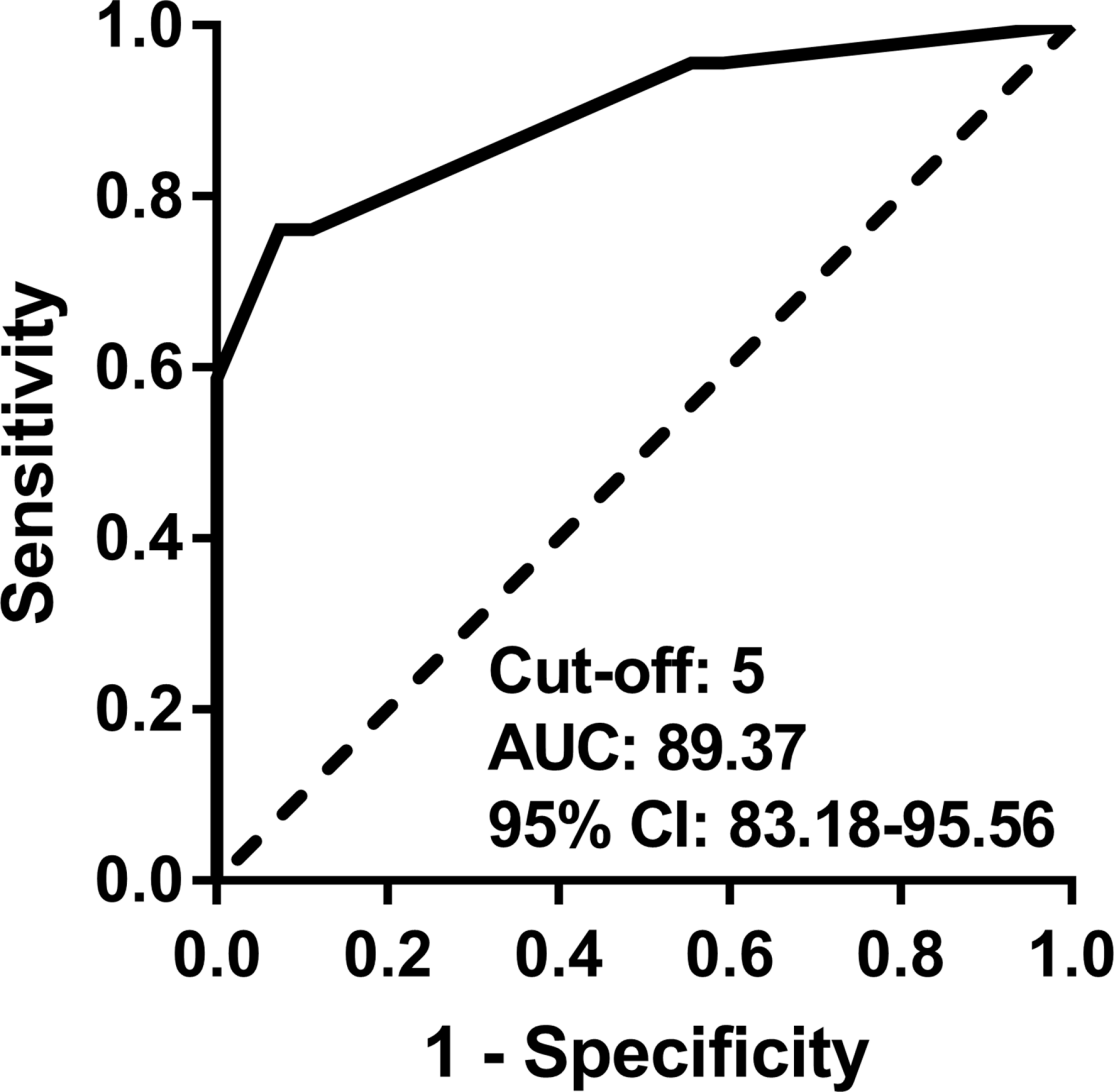
ROC curve for discriminating active disease and inactive disease using the new disease activity assessment tool. AUC, area under the curve; CI, confidence interval; ROC, receiver operating characteristic.

In the ROC curve analysis (**Figure 1**), the cut-off value in the new disease activity assessment tool that best discriminated active disease and inactive disease was 5 (≥5, active; <5, inactive). The AUC, sensitivity, specificity, accuracy, PPV, and NPV of the new disease activity assessment tool were 89.37 (95% CI 83.18–95.56), 76.09 (95% CI 63.76–88.42), 92.59 (95% CI 85.60–99.58), 85.00 (95% CI 78.00–92.00), 89.74 (95% CI 80.22–99.26), and 81.97 (95% CI 72.32–91.62), respectively (**Table 5**). The new disease activity assessment tool had significantly higher AUC and accuracy for differentiating active disease and inactive disease than NIH criteria and the ITAS2010, ITAS-ESR, and ITAS-CRP.

**Table 5:** Diagnostic performance of disease activity assessment tools in the derivation cohort.

	Cut-off	AUC (95% CI)	Sensitivity (95% CI)	Specificity (95% CI)	Accuracy (95% CI)	PPV (95% CI)	NPV (95% CI)
New assessment tool	≥5, active; <5, inactive	89.37 (83.18–95.56)	76.09 (63.76–88.42)	92.59 (85.60–99.58)	85.00 (78.00–92.00)	89.74 (80.22–99.26)	81.97 (72.32–91.62)
NIH criteria	≥2, active; <2, inactive	77.96 (69.95–85.96)*	54.35 (39.96–68.74)*	83.33 (73.39–93.27)*	70.00 (61.02–78.98)*	73.53 (58.70–88.36)*	68.18 (56.94–79.42)*
ITAS2010	≥2, active; <2, inactive	66.12 (55.64–76.61)*	65.22 (30.44–91.30)	62.96 (50.08–75.84)*	64.00 (54.59–73.41)*	60.00 (46.42–73.58)*	68.00 (55.07–80.93)*
ITAS-ESR	≥5, active; <5, inactive	75.58 (66.22–84.95)*	36.96 (23.01–50.91)*	88.89 (80.51–97.27)	65.00 (55.65–74.35)*	73.91 (55.96–91.86)	62.34 (51.52–73.16)*
ITAS-CRP	≥5, active; <5, inactive	71.34 (61.32–81.40)*	30.43 (17.13–43.73)*	92.59 (85.60–99.58)	64.00 (54.59–73.41)*	77.78 (58.57–96.99)	60.98 (50.42–71.54)*

AUC, Area under the curve; CI, confidence interval; CRP, C-reactive protein; ESR, erythrocyte sedimentation rate; ITAS2010, Indian Takayasu Clinical Activity Score; NIH, National Institute of Health; NPV, negative predictive value; PPV, positive predictive value.

*P < 0.05 compared with the new assessment tool.

### Validation of the New Disease Activity Assessment Tool

In the validation cohort, the mean patient age was 41.7 ( ± 15.5) years, and 82.6% of patients were female. The median ESR, CRP, NIH criteria, ITAS2010, ITAS-ESR, and ITAS-CRP values were 31.5 (17.0–83.3) mm/h, 3.4 (1.0–24.6) mg/L, 1.0 (1.0–2.0), 1.0 (0.0–3.0), 2.5 (2.0–4.3), and 2.0 (1.0–4.0), respectively. Proportions of patients with high ESR and high CRP were 67.4% and 39.1%, respectively. According to the PGA, 24 (52.2%) and 22 (47.8%) patients had active disease and inactive disease, respectively. Detailed characteristics of the patients in the validation cohort are shown in **Table 1**.

The diagnostic performance of the new disease activity assessment tool in the validation cohort was similar to that in the derivation cohort, with AUC, sensitivity, specificity, accuracy, PPV, and NPV of 85.23 (95% CI 74.37–96.09), 66.67 (95% CI 47.81–85.53), 95.45 (95% CI 86.74–104.16), 80.43 (95% CI 68.96–91.90), 94.12 (95% CI 82.94–105.30), and 72.41 (95% CI 56.14–88.68), respectively (**Table 6**).

**Table 6:** Diagnostic performance of the new disease activity assessment tool in the validation cohort.

AUC (95% CI)	Sensitivity (95% CI)	Specificity (95% CI)	Accuracy (95% CI)	PPV (95% CI)	NPV (95% CI)
85.23 (74.37–96.09)	66.67 (47.81–85.53)	95.45 (86.74–104.16)	80.43 (68.96–91.90)	94.12 (82.94–105.30)	72.41 (56.14–88.68)

AUC, area under the curve; CI, confidence interval; NPV, negative predictive value; PPV, positive predictive value.

## Discussion

In this study, we developed a new disease activity assessment tool derived from items obtained from NIH criteria and the ITAS2010. This new assessment tool had a higher accuracy in differentiating active disease and inactive disease than NIH criteria and the ITAS2010, ITAS-ESR, and ITAS-CRP. Given that accurate assessment of disease activity is important in therapeutic decision-making (18), this newly generated disease activity assessment tool with high accuracy has important clinical implications.

The ITAS2010 is a clinical assessment tool that comprehensively captures clinical manifestations for assessment of disease activity of TAK (13). The ITAS-ESR and ITAS-CRP additionally incorporate acute phase reactants (13). However, imaging findings are not included as a scoring item in the ITAS (13). As patients with active TAK commonly have non-specific disease manifestations and unreliable laboratory parameters, imaging findings are of importance in monitoring disease activity in patients with TAK (19, 20). On this basis, it is reasonable to combine clinical manifestations and acute phase reactants with imaging findings to yield a highly accurate disease activity assessment tool. Indeed, the new disease activity assessment tool consists of all the above-mentioned components (NIH criterion 1 and carotidynia for the clinical manifestation domain, high ESR for the acute phase reactant domain, and NIH criterion 4 for the imaging domain). Its diagnostic performance in discriminating active disease and inactive disease was significantly better than that of the ITAS2010, ITAS-ESR, and ITAS-CRP, which do not include imaging findings. These results reflect the importance of imaging findings in assessing disease activity of TAK.

NIH criteria include clinical manifestations, acute phase reactant (ESR), and imaging findings (3). Since an imaging finding (NIH criterion 4), is included as a criterion, NIH criteria (AUC, 77.96) had a higher AUC in discriminating active disease and inactive disease than the ITAS2010 (AUC, 66.12), ITAS-ESR (AUC, 75.58), and ITAS-CRP (AUC, 71.34), as could be expected. However, the AUC of NIH criteria for distinguishing active and inactive disease was lower compared with that of the new disease activity assessment tool (AUC, 89.37). This significantly higher AUC of the new assessment tool was striking, given that both disease activity assessment tools include items from all domains for assessing disease activity (clinical manifestations, acute phase reactants, and imaging findings). The difference in accuracy appears to stem from using a high ESR and carotidynia instead of NIH criterion 2 and NIH criterion 3, respectively, in the new assessment tool.

The fulfilment of NIH criterion 2 is defined as new or worsening of elevated ESR (3). An important limitation of this definition is that it does not fully reflect the current absolute state of acute phase reactant. For instance, if the ESR was 90 mm/h previously and is 72 mm/h currently, NIH criterion 2 will be considered as not being met, even though the current ESR of 72 mm/h is still high (i.e. false negative). On the other hand, the item “high ESR” included in the new disease activity assessment tool captures elevated ESR regardless of the previous ESR, and therefore reflects the current absolute state better than NIH criterion 2. Hence, using a high ESR instead of NIH criterion 2 could have attributed to a higher sensitivity (a lower chance of a false negative) in the new disease activity assessment tool (sensitivity, 76.09) than that of NIH criteria (sensitivity, 54.35).

Another difference between the new disease activity assessment tool and NIH criteria is the use of carotidynia instead of NIH criterion 3. The fulfilment of NIH criterion 3 is defined as new or worsening of ischaemia [claudication, diminished or absent pulse, bruit, vascular pain (carotidynia), and asymmetric blood pressure] (3). Of symptoms included in this criterion, carotidynia usually occurs as a direct result of active vessel inflammation, whereas the other symptoms usually occur as a result of irreversible structural damage of the vessel from previous inflammation, rather than active vessel inflammation (3). Therefore, inclusion of vascular symptoms other than carotidynia could result in a false positive detection of active disease. In the new disease activity assessment tool, carotidynia is used as the only item reflecting vascular symptoms, which may have attributed to a higher specificity (a lower chance of a false positive) in the new disease activity assessment tool (specificity, 92.59) than in NIH criteria (specificity, 83.33).

The new disease activity assessment tool weights each item based on simplified β coefficients. The weights varied among items, with NIH criterion 4 (simplified β coefficient: 4) having the highest weight and NIH criterion 1 (simplified β coefficient: 2) having the lowest weight. It should be noted that fulfilment of one item only is not sufficient to classify a patient as having active disease (the cut-off value for active disease was ≥5). On the other hand, patients with any combination of two items results in a score of ≥5, and such patients will be classified as having active disease. Therefore, the new disease activity assessment tool can be simplified similar to NIH criteria as follows: fulfilment of two or more of the following features: (i) high ESR, (ii) NIH criterion 1, (iii) NIH criterion 4, and (iv) carotidynia.

This study had several limitations. First, histopathology of the artery, which is the true gold standard for assessing disease activity (18), was not available. We instead used the PGA as the gold standard. As the treating physician was aware of all clinical information including clinical manifestations, laboratory test parameters, and imaging findings, we assumed that the PGA was most suitable for use as a gold standard for validating the disease activity assessment tool. Indeed, the PGA is widely used as a disease activity comparator (11, 13, 14, 21). Second, we lacked data on ^18^F-fluorodeoxyglucose positron emission tomography/CT (^18^F-FDG PET/CT) scan results, which are useful in assessing disease activity of TAK (22–24). If these data had been present and used in combination with the items from current disease activity assessment tools, they might have yielded a new disease activity assessment tool with even higher accuracy. However, ^18^F-FDG PET/CT scans are expensive and have limited availability; therefore, their value in routine clinical practice is limited. CT scans, on the other hand, are less expensive and easily accessible compared with ^18^F-FDG PET/CT scans. Therefore, our results could be more applicable to routine clinical practice setting. Moreover, the accuracy of the new disease activity assessment tool we developed here in the absence of these data was still good (AUC >0.8) (25), and is therefore clinically meaningful. Third, we lack longitudinal data and were unable to assess response to change of the disease activity assessment tool as a reflection of therapy.

In conclusion, we developed a new disease activity assessment tool that consists of high ESR (3 points), NIH criterion 1 (2 points), NIH criterion 4 (4 points), and carotidynia (3 points) (total score of ≥5 indicates active disease, or simply, fulfilment of ≥2 components indicates active disease), which has a higher accuracy in discriminating active disease and inactive disease than the currently used clinical assessment tools. This new disease activity assessment tool is easy to perform and could be useful for more accurately classifying patients with TAK into active TAK and inactive TAK.

## Data Availability Statement

The original contributions presented in the study are included in the article/supplementary material. Further inquiries can be directed to the corresponding author.

## Ethics Statement

The studies involving human participants were reviewed and approved by the Institutional Review Board (IRB) of Gangnam Severance Hospital (IRB No: 3-2021-0445). The ethics committee waived the requirement of written informed consent for participation.

## Author Contributions

OCK and M-CP contributed to the conception and design of the study. OCK and M-CP participated in acquisition of data, data analyses and data interpretation. OCK and M-CP wrote the manuscript. All authors contributed to the article and approved the submitted version.

## Funding

This study was supported by a faculty research grant of Yonsei University College of Medicine (6-2019-0111).

## Conflict of Interest

The authors declare that the research was conducted in the absence of any commercial or financial relationships that could be construed as a potential conflict of interest.

## Publisher’s Note

All claims expressed in this article are solely those of the authors and do not necessarily represent those of their affiliated organizations, or those of the publisher, the editors and the reviewers. Any product that may be evaluated in this article, or claim that may be made by its manufacturer, is not guaranteed or endorsed by the publisher.
